# A biomimetic membrane platform for predictive screening and rational engineering of endosomally escaped lipid nanoparticle-CpG oligodeoxynucleotides delivery systems

**DOI:** 10.7717/peerj.21147

**Published:** 2026-05-05

**Authors:** Wenyan Cui, Yongjian Cheng, Wenzhong Chen, Behloul Nouredine, Jing He, Yan Zuo, Yue Yang

**Affiliations:** 1 Department of Pharmaceutical Analysis and R&D, Nanjing Geneleap Biotechnology Co., Ltd., Nanjing, Jiangsu, China; 2 State Key Laboratory of Advanced Drug Delivery and Release Systems, Shandong Luye Pharmaceutical Co., Ltd., Yantai, Shandong, China

**Keywords:** LNP-CpG ODN, Biomimetic membrane, Endosomal escape, TLR9, Delivery system optimization

## Abstract

The efficient delivery of CpG oligodeoxynucleotides (CpG ODN) adjuvants is constrained by the limited endosomal escape capability of lipid nanoparticles (LNPs). To evaluate this critical parameter, we constructed an *in vitro* escape evaluation platform that integrates serum protein corona simulation, a biomimetic phospholipid bilayer barrier, and pH regulation. This platform enables quantitative analysis of the transmembrane delivery mechanism of LNP-CpG ODN while overcoming limitations of cellular models for studying carrier-biointerface interactions. Through optimization of the biomimetic membrane formulation, an optimal balance between high vesicle stability (PDI < 0.18) and membrane fusion activity was achieved. Furthermore, a safer and more efficient preparation workflow was established by replacing chloroform with dichloromethane and eliminating the vacuum-drying step. Importantly, the results reported here identify the pH 6.0 microenvironment, which simulates late endosomal conditions, as the critical functional window for escape, with its efficiency showing a significant positive correlation with TLR9 pathway activation intensity (*r* = 0.8978, *p* < 0.0001). Overall, this work establishes a dual-parameter correlation framework linking escape efficiency to immune activity, providing a new paradigm for the rational design and optimization of LNP delivery systems.

## Introduction

CpG oligodeoxynucleotides (CpG ODN) are immunostimulatory sequences centered around unmethylated CpG motifs, inducing innate immune responses through activation of Toll-like receptor 9 (TLR9), thereby enhancing adaptive immune responses (Zhang et al., 2023; Wang et al., 2024). Based on chemical structure differences, CpG ODN sequences are classified into three types: A, B, and C. Type B, with a phosphorothioate backbone prolonging *in vivo* half-life, primarily activates B cells. Type A readily forms multimeric/nanoparticle structures, posing challenges for *in vivo* delivery, often requiring strategies such as virus-like particle (VLP) encapsulation. Type C combines the activity of A and B types, simultaneously activating plasmacytoid dendritic cells (pDCs) and B cells (Shirota & Klinman, 2014; Katsuda et al., 2011; Li et al., 2020). In clinical applications, CpG ODN demonstrates unique value as a vaccine adjuvant and in tumor immunotherapy. For instance, the FDA-approved HEPLISAV-B hepatitis B vaccine, by incorporating CpG-1018, achieves higher antibody titers with fewer doses (Awad et al., 2021). Additionally, in tumor immunotherapy, CpG ODN significantly enhances CD8+ T cell activity and antibody responses through physical combination, covalent conjugation, co-delivery, or strategic combinations (*e.g.*, local injection) with tumor antigens, exhibiting broad application potential (Dasari et al., 2023; Wilson et al., 2013; Zeng et al., 2025). However, the efficacy of CpG ODN is highly dependent on its subcellular localization—free CpG ODN is susceptible to degradation by serum nucleases and requires precise delivery to the endosomal compartments of immune cells to activate TLR9 signaling (Chen et al., 2019). This characteristic presents a dual challenge for delivery systems: achieving efficient intracellular delivery while avoiding premature release of nucleic acids into the cytosol, which leads to TLR9 signal inactivation or activation of unintended DNA sensing pathways.

Lipid nanoparticles (LNPs), as representative non-viral delivery systems, have continuously unleashed revolutionary potential in nucleic acid drug development, through component engineering and innovations in delivery mechanisms. Compared to traditional delivery systems, LNPs not only exhibit excellent biocompatibility, high encapsulation efficiency, and strong nuclease resistance, but also enable exploration of tissue-specific targeted delivery through surface functionalization (Bian et al., 2025; Wang et al., 2025; Dilliard, Cheng & Siegwart, 2021; Yan et al., 2025). These advantages have been validated by several milestone drugs: in the vaccine field, LNP-based mRNA vaccines (*e.g.*, Pfizer/BioNTech and Moderna COVID-19 vaccines) activate potent immune responses by delivering mRNA encoding viral spike proteins to the cytosol (Vogel et al., 2021; Corbett et al., 2020); in gene therapy, the world’s first approved LNP-delivered siRNA drug, Patisiran (trade name Onpattro), specifically silences transthyretin (TTR) expression in the liver by delivering siRNA to hepatocytes, and thus successfully treating hereditary transthyretin amyloidosis (hATTR) (Adams et al., 2018; Maurer et al., 2023), and the same time, marking a clinical breakthrough for LNP technology in RNA interference therapy. Despite these advances, improving further the delivery efficiency, especially for non-liver or cell type-specific targeting, still faces a core challenge—the limitation of endosomal escape efficiency (Bader, Brigger & Leroux, 2024; Albertsen et al., 2022). This limitation underscores the necessity of deeply understanding and optimizing the endosomal escape mechanism, which is a core objective for engineering utilizing the modular design advantages of LNPs.

Endosomal escape efficiency is a key determinant of successful LNP-mediated delivery. Following the cellular uptake of LNPs *via* endocytosis, it is necessary to ensure that CpG ODN is delivered and retained in TLR9-rich early endosomal compartments to avoid its premature or complete escape into the cytosol. This imposes special requirements on LNP behavior for CpG ODN delivery (Niidome & Huang, 2002; Hazeki et al., 2011). Previous studies have already shown that conventional LNP formulations often face low efficiency in endosomal delivery due to mismatched protonation kinetics of ionizable lipids and endosomal acidification processes (Gilleron et al., 2013; Liu et al., 2024). This mismatch can result in CpG ODN failing to adequately reach TLR9-containing compartments, therefore, significantly reducing its immunostimulatory efficacy and potentially triggering unintended inflammatory responses due to cytosolic leakage. In recent years, research has aimed to coordinate the spatiotemporal escape characteristics and subcellular localization requirements of LNPs by developing novel ionizable lipids (Bian et al., 2025; De Lombaerde et al., 2024), for instance, designing acid-responsive lipid materials (*e.g.*, lipids containing hydrazone bonds or specific pH-sensitive groups) (Sato et al., 2020; Abd Elwakil et al., 2019), or incorporating cell-targeting ligands (*e.g.*, mannose modification for targeting antigen-presenting cells) (Fei et al., 2024; Ding et al., 2025). Simultaneously, *in vitro* detection methods are continuously developed and/or optimized to quantitatively evaluate escape efficiency: Fluorescence resonance energy transfer (FRET) technology enables real-time monitoring of nucleic acid dissociation dynamics from LNP carriers (Sahay et al., 2013; Ma et al., 2024); artificial endosome models assess LNP membrane fusion or disruption capabilities by simulating pH gradients (*e.g.*, from pH 7.4 to 5.0) (Aliakbarinodehi et al., 2024); TLR9 pathway activation efficiency detection (*e.g.*, ELISA quantification of IFN-*α*) is used to verify the functional localization of CpG ODN (Sivori et al., 2010; Reimer et al., 2013). However, these *in vitro* evaluation systems face an important limitation: most methods fail to systematically simulate the coupled effects of two critical physiological barriers—“plasma protein corona formation” and “dynamic evolution of the endosomal acidification microenvironment”. Consequently, they struggle to accurately assess the interfacial behavior and transmembrane delivery efficiency of LNPs under the influence of protein corona. This limitation constitutes a core challenge in the current optimization of LNP-CpG ODN delivery systems. Therefore, this study aims to construct a biomimetic membrane evaluation system that integrates serum protein corona simulation with endosomal pH gradient regulation. This system addresses the physiological relevance gap in existing methods and provides a reliable, standardized experimental basis for the quantitative analysis of LNP-CpG ODN escape behavior.

Herein, an *in vitro* escape evaluation model for LNP-CpG ODN drugs has been constructed based on a biomimetic cell membrane. In the experimental design, the key initial process of plasma protein adsorption forming a protein corona after the LNP-CpG ODN drug enters in contact with body fluids *in vivo* was first simulated by pre-binding the LNP-CpG ODN drug with serum (Xiao et al., 2023). Subsequently, a biomimetic membrane barrier with a phospholipid bilayer structure (core-simulating endosomal membrane) is introduced. The system is then gradually adjusted to an endosomal acidic microenvironment through pH regulation, thereby critically simulating the dynamic evolution of the protein corona formation and endosomal acidification microenvironment that LNPs experience after cellular endocytosis. During this process, highly sensitive nucleic acid dyes are used for *in situ* capture and fluorescent quantification of free CpG ODN that successfully traverse the biomimetic membrane barrier, achieving precise evaluation of LNP *in vitro* escape efficiency. This method innovatively integrates serum protein corona simulation, biomimetic membrane barrier construction, pH gradient regulation, and quantitative detection technology. It provides a simplified and controllable platform compared to complex cellular systems, that facilitates independent and quantitative study of carrier-membrane interface interactions and transmembrane delivery kinetics under the influence of the protein corona. This model provides a new tool for optimizing the delivery efficiency of nucleic acid drugs, their preclinical evaluation, as well as key experimental evidence and theoretical support for developing a standardized *in vitro* systems for elucidating the intricacies surrounding the fate of LNP drugs within the cell.

## Experimental Section

### Materials & Methods

1,2-dioleoyl-sn-glycero-3-phosphocholine (DOPC), 1,2-dioleoyl-sn-glycero-3-phospho-L-serine sodium (DOPS), 1,2-dioleoyl-sn-glycero-3-phosphoethanolamine (DOPE) were obtained from Avanti Polar Lipids (Alabaster, AL, USA). PBS was obtained from Gibco. C57BL/6 mouse serum was obtained from IPHASE Co., China. SYBR Gold Dye was purchased from Thermo Fischer Scientific. Triton X-100, chloroform, and dichloromethane were obtained from Aladdin Scientific. hPBMC cells were purchased from SAILYBio Co., Shanghai, China. All chemicals and solvents used were of reagent grade and employed without further purification.

Rotary Evaporator was purchased from Hei-Vap Co., China. Particle size analyzer was purchased from Malvern Panalytical. Microplate reader was purchased from Biotek. Gas chromatograph was purchased from Agilent. The LNP-CpG ODN samples used in this study were provided by the Drug Delivery R&D Department of Nanjing Geneleap Biotechnology Co., Ltd. The LNP formulation was composed of ionizable lipid GLL001, DSPC, cholesterol, and mPEG-DMG-2K at a molar ratio of 50:10:38.5:1.5.

**Cell culture and ethical statement.** Frozen human peripheral-blood mononuclear cells (PBMC) from 25 year-old healthy Caucasian male donor were purchased from Shanghai Saili Biopharmaceutical Co., Ltd., Shanghai, China (catalog number XFB-HP100B, Lot # SC12334W) and processed in accordance with the supplier’s instructions. The vendor confirmed IRB approval and informed consent.

### Construction and process optimization of biomimetic cell membrane

#### Preparation method of biomimetic cell membrane

The preparation of the biomimetic cell membrane employed a phospholipid bilayer to simulate the basic structure of the natural cell membrane, using DOPS, DOPE, and DOPC as the main components to construct the membrane skeleton. Specifically, the negative charge characteristic of DOPS simulates the charge environment of the endosomal membrane, while DOPE and DOPC reproduce the biophysical properties and dynamic structural characteristics of the endosomal membrane by regulating membrane fluidity and phase transition properties. The specific preparation procedure was as follows: First, DOPS, DOPE, and DOPC were dissolved at predetermined molar ratios, then transferred to a pear-shaped flask. Subsequently, a rotary evaporator was used under reduced pressure in a water bath temperature to completely evaporate the solvent and form a uniform lipid film. The pear-shaped flask coated with the lipid film was placed in a vacuum desiccator for further drying and to completely remove residual organic solvents. After drying, phosphate-buffered saline (PBS, pH 7.4) was added and gently agitated to promote lipid film hydration and peeling, and forming a multilamellar vesicle suspension. Finally, repeated extrusion homogenizations using a mini-extruder (*e.g.*, with polycarbonate membranes, pore size 400 nm) were performed to obtain biomimetic cell membrane vesicles of uniform particle size. Finally, the preparations were stored at 4 °C until further use. The average particle size and PDI were determined by dynamic light scattering (DLS), and the vesicular morphology was further characterized by cryo-electron microscopy (Cryo-EM) to ensure membrane structural homogeneity and stability.

#### Optimization of key parameters in preparation process

##### Lipid composition screening and evaluation.

The bilayer structure of the cell membrane is mainly composed of phosphatidylcholine (PC), phosphatidylethanolamine (PE), and anionic lipids (*e.g.*, phosphatidylserine, PS), all in specific physiological ratios (Zhukov & Popov, 2023; Sezgin et al., 2017). PE (*e.g.*, DOPE), due to its conical molecular structure and ability to promote membrane curvature and fusion, is crucial for membrane dynamic reorganization (*e.g.*, endosomal escape); while PC (*e.g.*, DOPC) provides membrane stability as a structural scaffold, and anionic lipids (*e.g.*, DOPS) participate in charge-mediated interactions. In natural cell membranes, PE can account for up to 25–30%, significantly higher than PS (<10%) (Xu et al., 2013; Casares, Escribá & Rosselló, 2019). Based on this, two core formulations were designed in this study: 1:8:1 ratio of DOPS, DOPC, and DOPE respectively, to simulate a high-stability membrane environment, while a 1:1:2 ratio of the same components was formulated to simulate a closer PE physiological abundance that enhances membrane remodeling potential (Zhang et al., 2014; Aliakbarinodehi et al., 2022; Zhang, 2019). A control formulation of only DOPS and DOPC lacking DOPE (1:8 ratio, respectively) was also prepared to analyze the functional necessity of DOPE. A systematic comparison of the characteristics of the different formulations (particle size homogeneity, content accuracy, structural integrity) and functional performance (escape efficiency reproducibility, storage stability) was conducted to screen for the optimal ratio that fulfills the dual criteria of biomimetic properties and technical feasibility.

##### Strategy for solvent system substitution.

In the preparation of anionic formulation solutions, chloroform is widely adopted due to its solvent properties. However, chloroform, as a regulated reagent, is difficult to obtain, possesses significant hepatorenal toxicity, and potential carcinogenic risk, limiting thus the safety and convenience of its routine use. To overcome this limitation, this study proposes using dichloromethane as an alternative solvent. Dichloromethane is not only more readily available but also exhibits relatively lower toxicity than chloroform and similar physicochemical properties (*e.g.*, polarity, boiling point). Taken together, dichloromethane presents a solid theoretical potential for substituting chloroform. Accordingly, the feasibility of replacing chloroform with dichloromethane was systematically evaluated, focusing on comparing their dissolution capacity for the same formulation, lipid content recovery, and differences in LNP escape efficiency to confirm equivalence.

##### Verification of the necessity of the vacuum drying step.

In the preparation process of anionic spherical membranes, the literature generally emphasizes the prolonged vacuum drying steps (typically hours to overnight) to completely remove residual organic solvents (Zhang et al., 2014; Goodchild et al., 2014). Although this step aims to ensure membrane quality and safety, it significantly increases operational complexity and the overall preparation time/cost, becoming a bottleneck for process optimization. To address this issue, different vacuum drying times (0 h, 1 h, 3 h, 6 h) were analyzed in terms of dichloromethane solvent volatility. and designed. To precisely quantify the residual dichloromethane levels in the membranes at each time point, high-sensitivity headspace sampling-gas chromatography (HS-GC) was used for quantitative analysis. The specific method was as follows: Agilent 8890 gas chromatograph equipped with Agilent 8697 headspace sampler was used, and Agilent DB-624 column (30 m × 530 µm × 3 µm) was selected. As for the chromatographic conditions: injector temperature 250 °C, split ratio 1:1, injection volume 1,000 µL, carrier gas (N_2_) flow rate 3.0 mL/min; Detector (FID) temperature 250 °C, hydrogen flow 30 mL/min, air flow 400 mL/min; oven temperature program: initial 50 °C hold for 6 min, ramp at 50 °C/min to 200 °C hold for 3 min; headspace conditions: equilibration temperature 85 °C, equilibration time 20 min, loop temperature 100 °C, transfer line temperature 110 °C.

This method was established to evaluate the actual minimum required drying time to streamline the process, improve efficiency, while strictly ensuring that the solvent residue in the final product meets safety standards.

### Establishment and evaluation of the *in vitro* escape evaluation system

#### Quantitative detection procedure for escape efficiency

In this experiment, a fluorescence-based quantitative detection system was constructed by simulating the interaction between lipid nanoparticles (LNP) and serum proteins in the physiological environment as well as membrane escape behavior. The biomimetic cell membrane, simulating the endosomal membrane in cell, mediates initial binding with cationic lipids on the LNP surface *via* electrostatic interactions. The acidic pH environment, simulating endosomal lumen (pH 5.0−6.5) triggers conformational changes in LNP lipid components, inducing fusion between the LNP and the biomimetic membrane, which in turn leads to the release of CpG ODN (Aliakbarinodehi et al., 2022). By introducing SYBR Gold nucleic acid dye into the system, it specifically binds to the free nucleic acids present outside of the biomimetic membrane, and the quantitative measurement of the fluorescence intensity allows a direct measurement of the efficiency of nucleic acid escape from the LNP. The specific steps are as follows: First, to simulate *in vivo* protein corona formation, LNPs were mixed with an equal volume of non-heat-inactivated C57BL/6 mouse serum (final concentration 50%, v/v) and incubated at 37 °C for 20 min. Subsequently, two sets of treatment systems were prepared separately: (1) LNP-serum-anion mixture where anionic lipids were added at an anionic/cationic lipid molar ratio of 1, and PBS (pH 6.0) was used to adjust the system pH to 6.0; (2) LNP-serum-Triton mixture where an equal volume of 1% Triton solution was added as a complete lysis control. All samples were incubated at 37 °C for 15 min. Simultaneously, an empty LNP-serum-PBS (pH 6.0) mixture was prepared as a negative control; and gradient-diluted CpG ODN alone was prepared as reference standard for quantitative detection. Next, 100 µL of the prepared samples were transferred into a black 96-well plate (all samples were analyzed in triplicates), and 100 µL of SYBR Gold dye diluted 10,000-fold (diluted in TE buffer) was added into the wells. The plates were then incubated at 25 °C in the dark with constant shaking at 100 rpm for 15 min to complete nucleic acid-dye binding. Finally, the fluorescence signal was detected using a microplate reader (excitation wavelength 495 nm, emission wavelength 537 nm). For result analysis, the fluorescence intensity was converted into nucleic acid concentration based on the CpG ODN standard curve; using the concentration corresponding to the fluorescence value of the Triton group as the 100% release benchmark and the PBS group as the background signal, the escape efficiency was calculated using the following formula: Escape efficiency (%) = ((Concentration of LNP-serum-anion mixture group)–(Concentration of LNP-serum-PBS mixture group))/((Concentration of LNP-serum-Triton mixture group)–(Concentration of LNP-serum-PBS mixture group)) ×100%.

#### Functional simulation of endosomal acidification microenvironment

The endosomal escape behavior of lipid nanoparticles (LNPs) is inherently a pH-dependent cascade process, precisely mapping the acidification dynamics of cellular endocytic transport (Spadea et al., 2022; Sayers et al., 2019). Under physiological pH 7.4 conditions, LNPs are electrically neutral and initially enter cells *via* passive fusion or endocytosis. As endosomes mature and acidify to the critical threshold pH of 6.0, the amine groups of ionizable lipids within the LNPs become protonated, conferring a net positive charge to the particle surface. This charge reversal triggers electrostatic interactions with anionic lipids (*e.g.*, phosphatidylserine) in the endosomal membrane, causing membrane destabilization and LNP structural rearrangement. This primarily manifests as dissociation of the lipid bilayer, thereby efficiently releasing the encapsulated active substance. This process is kinetically rapid (seconds to minutes). When acidification continues to the lysosomal stage (pH ∼ 5.0), the strongly acidic environment not only inhibits charge-mediated membrane interactions but also activates lysosomal hydrolases (*e.g.*, cathepsins), leading to irreversible degradation of the LNP and its cargo, leading to an escape efficiency quasi-null (Gilleron et al., 2013; Paramasivam et al., 2022; Chatterjee et al., 2024). Therefore, to comprehensively validate this mechanism and ensure methodological accuracy, the differences in LNP-CpG ODN escape efficiency at different pH nodes (7.4, 6.0, 5.0) was evaluated.

#### Correlation study between escape efficiency and biological activity

The LNP samples involved in this study were all prepared using microfluidic technology, their composition includes four lipids (ionizable lipid/phospholipid/cholesterol/PEG lipid) and the nucleic acid cargo CpG ODN, strictly adhering to established molar ratios. For LNP samples prepared by different processes, their endosomal escape efficiency was quantitatively assessed using the method established above. Simultaneously, their biological activity was detected as follows: PBMC were thawed according to the supplier’s instructions, and the cell viability was confirmed to be above 95%. Next, the cells were resuspended in 1,640 culture medium supplemented with 10% fetal bovine serum, and 5  ×  10^5^ cells were seeded into 96-well plate wells. Then, LNP-CpG ODN samples were added into the well to a final concentration of CpG ODN of 1 µM (total volume 200 µL), negative control wells were left untreated (only PBMC in culture medium). All samples were assayed in triplicates. The plates were incubated for 24 h at 37 C and 5% CO_2_. The next day, cell culture supernatants were collected and centrifuged at 400 × g for 10 min to remove all cell debris, and the levels of IFN*α* was measured using a VeriKine Human IFN*α* ELISA Kit (Cat #41100; PBL Assay Science, Piscataway, NJ, USA) according to the manufacturer’s instructions.

Subsequently, correlation analysis was performed between the endosomal escape efficiency data and the ELISA-determined IFN-*α* concentration (biological activity) for the same batch of LNP samples from different processes to calculate the correlation between the two.

## Results

### Results of biomimetic cell membrane preparation process optimization

#### Lipid composition-dependent performance characterization

##### Characterization of biomimetic cell membrane size and homogeneity.

**Figure 1:**
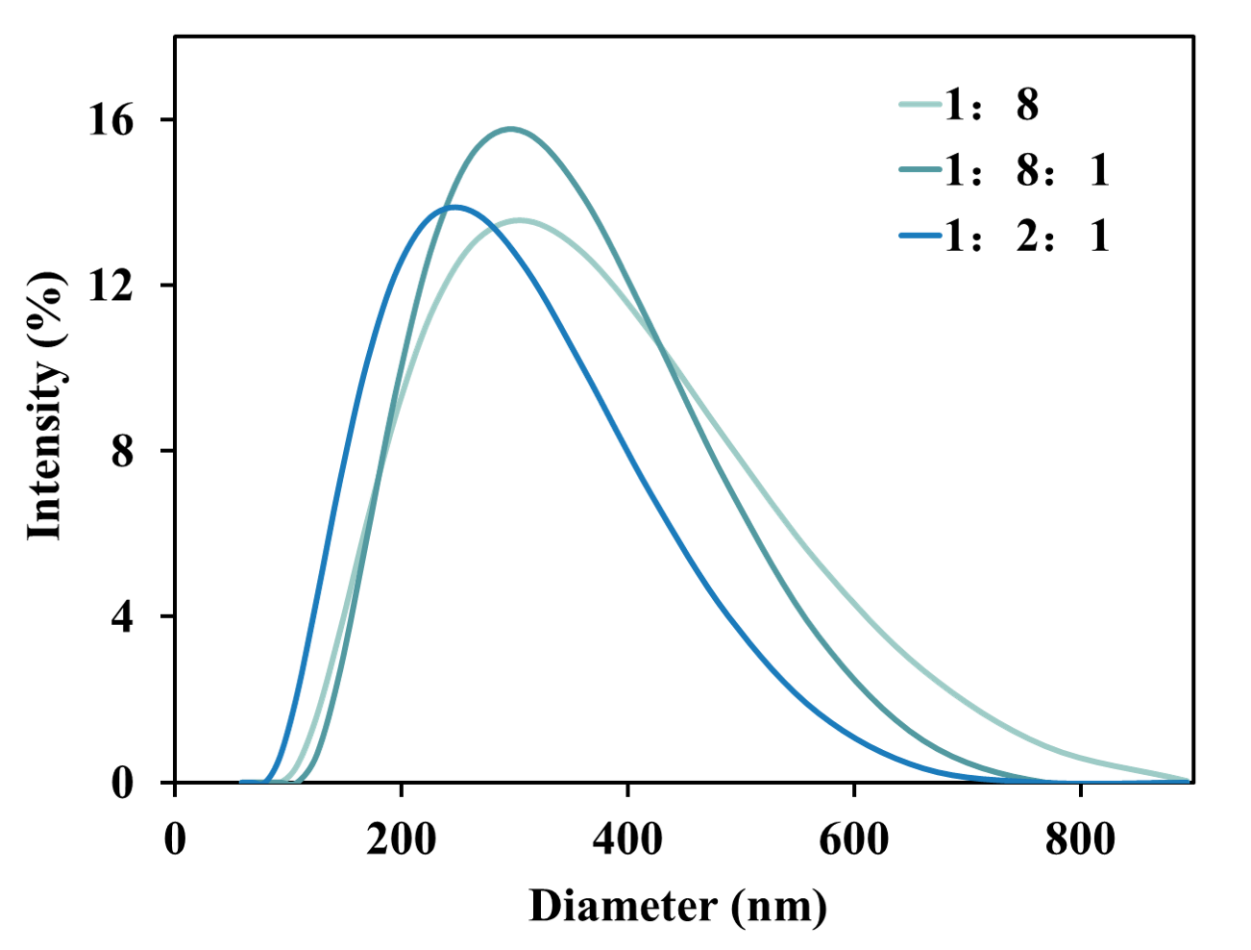
Size distribution of biomimetic cell membrane vesicles with different lipid compositions.

Three types of biomimetic membranes were prepared with different phospholipid compositions *via* the thin-film hydration method, with the core objective of constructing an *in vitro* model capable of effectively predicting LNP endosomal escape behavior. As shown in Fig. 1, all biomimetic membranes exhibited good monodispersity (PDI < 0.18). When the the formulation was composed of DOPS, DOPC and DOPE at ratio of 1:8:1, structurally stable benchmark vesicles with an average particle size of 257.8 nm were formed. Removal of the DOPE component (DOPS:DOPC at 1:8 ratio) led to an increase in particle size to 268.2 nm, suggesting potential impairment of membrane stability. Adjustment to the physiologically simulated ratio 1:1:2 resulted in a significant reduction in vesicle size to 217.3 nm, indicating that the high proportion of negatively charged DOPS and conical DOPE molecules promoted tighter lipid packing, forming membranes with higher curvature and more compact structures.

Based on this, these differences in physical properties were thought to directly determine the model’s predictive capability. The smaller size directly results in higher membrane curvature and a larger specific surface area, which not only more accurately simulates the dynamic structure of intracellular endosomal membranes *in vivo* but also significantly increases the contact probability and adsorption efficiency between LNPs and the membrane, which is prerequisite for triggering subsequent membrane fusion or disruption. Furthermore, the lower PDI ensures high consistency in the membrane barrier encountered by LNPs in each experimental run. This minimizes data variability caused by inherent vesicle property fluctuations, allowing the measured differences in escape efficiency to reflect more accurately the functional characteristics of the LNPs themselves, thereby significantly enhancing the model’s reliability and predictive value in discriminating between different formulations.

##### Comparison of escape efficiency under different formulations.

**Figure 2:**
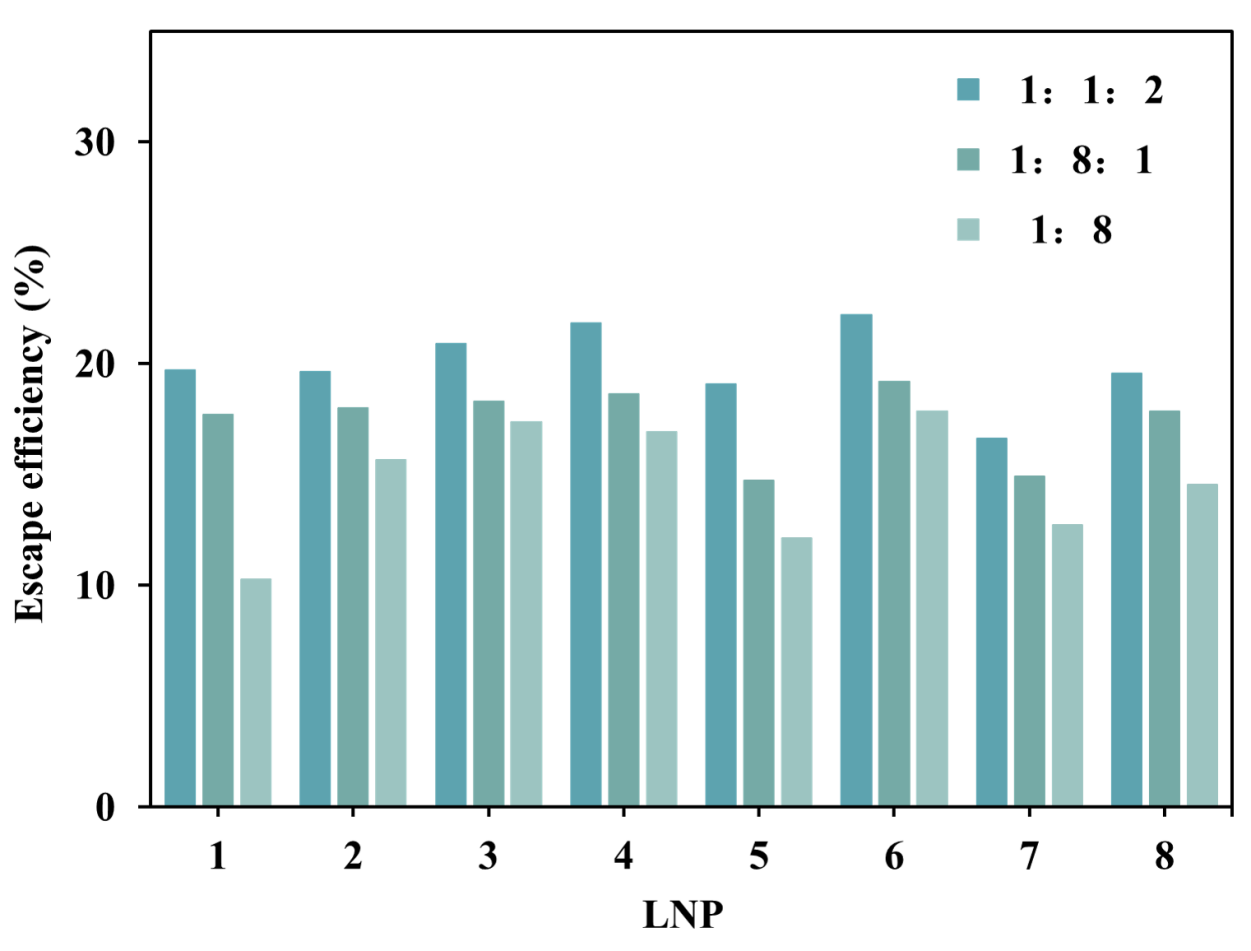
Comparison of LNP-CpG escape effciency using biomimetic membranes with different lipid compositions (*n* = 1).

As shown in Fig. 2, across eight independent biological samples, the three lipid formulations exhibited a universal delivery pattern: the 1:1:2 formulation consistently demonstrated the highest escape efficiency, clearly superior to 1:8:1 and 1:8 formulations. Crucially, all samples strictly adhered to the efficiency hierarchy: 1:1:2 > 1:8:1 > 1:8, confirming the dose-dependent enhancement effect of DOPE. This trend also proves that the high-DOPE group (1:1:2), due to its smaller vesicle diameter and high curvature structure, promotes endosomal membrane fusion, while the DOPE-deficient group (1:8) suffers from membrane structural instability (local fusion phenomena) leading to reduced sample escape efficiency.

##### Cryo-electron microscopy validation of membrane structure.

**Figure 3:**
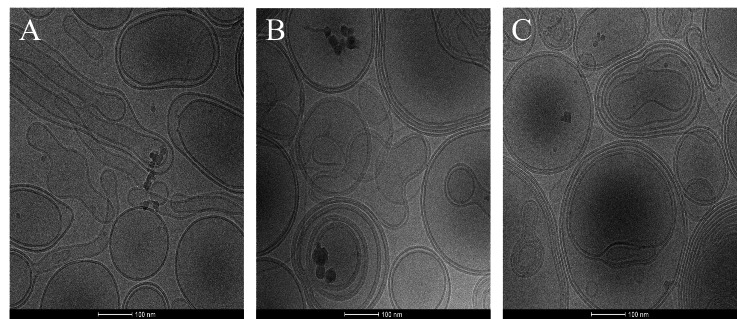
Cryo-electron microscopy analysis of three biomimetic cell membrane formulations. (A) Formulation A (1:1:2) induced intense negative curvature stress, driving lipid molecules to self-assemble into tubular networks and semi-open lipid lamellae. (B) Formulation B (1:8:1) formed unilamellar vesicles with excellent structural stability, it exhibited inadequate responsiveness to membrane remodeling. (C) Formulation C (1:8:0) generated multilamellar concentric structures due to the absence of DOPE, with further impairing fusion activity.

To construct a biomimetic cell membrane platform for evaluating LNP endosomal escape efficiency, the mesostructure-function correlation of three anionic lipid formulations was determined using cryo-electron microscopy (cryo-EM). Results demonstrated that the high proportion of DOPE (50 mol%) in Formulation A (1:1:2) induced intense negative curvature stress, driving lipid molecules to self-assemble into tubular networks and semi-open lipid lamellae (Fig. 3A), significantly enhancing membrane dynamic remodeling capacity. Coupled with the negatively charged microenvironment provided by DOPS (simulating endosomal membrane charge characteristics), this system synergistically reduced the membrane fusion energy barrier through charge pairing under acidic pH, thereby efficiently amplifying LNP-triggered transient pore formation and cargo release signals, which is a property closely aligned with the core mechanism of endosomal escape in nucleic acid delivery in a cell. By contrast, while Formulation B (1:8:1) formed unilamellar vesicles with excellent structural stability, it exhibited inadequate responsiveness to membrane remodeling (Fig. 3B). Formulation C (1:8:0) generated multilamellar concentric structures due to the absence of DOPE, with further impairing fusion activity (Fig. 3C). Based on these findings, Formulation A (1:1:2) was selected as the optimal biomimetic membrane composition for establishing a detection platform capable of quantifying LNP escape efficiency while maintaining biomimetic relevance.

##### Long-term stability assessment (4 °C).

During the stability assessment, ultra-low temperatures (−20 °C/−80 °C) caused destructive effects on biomimetic cell membranes of all formulations, manifested as severe precipitation and bimodal particle size distribution. This is primarily attributed to the synergistic effect of mechanical stress from ice crystal formation and low-temperature phase transitions of lipids (*e.g.*, propensity for inverse hexagonal phase in DOPE) disrupting biomimetic membrane integrity. However, under long-term storage conditions at 4 °C, the three formulations exhibited significant differences in stability (Table 1). The 1:1:2 formulation demonstrated an excellent long term stability. This could be explained by the precise functional balance of its lipid composition, where high proportion DOPE provides necessary membrane curvature stress regulation through its conical molecular structure, DOPS provides negative charge to effectively inhibit particle aggregation *via* electrostatic repulsion, and both synergistically assembled with DOPC to maintain the whole system’s thermodynamic steady state. After 6 months of storage, particle size maintained a monomodal distribution (224 nm → 209.6 nm, fluctuation < 7%), with no occurrence of aggregation or phase separation. By contrast, the 1:8:1 formulation showed poorer stability, exhibiting a bimodal distribution after 6 months at 4 °C, indicating progressive lipid phase separation. The 1:8 formulation had the worst stability, showing a bimodal distribution after only 15 days at 4 °C, indicating a severe defect in colloidal stability. The instability of these two formulations was mainly caused by compositional imbalance where insufficient or absence of DOPE led to loss of membrane curvature stability; low DOPS percentage rendered electrostatic shielding ineffective; and excessive DOPC enhanced membrane rigidity. Such conditions were detrimental to the dynamic equilibrium, thereby triggering lipid phase separation (bimodal particle size distribution).

**Table 1:** Particle size (nm) trend over time under storage at 4 °C.

**Formulation group**	**0 days**	**15 days**	**2 months**	**6 months**
1:1:2	224.0	215.3	234.6	209.6
1:8:1	261.0	256.3	280.0	Bimodal
1:8	268.2	Bimodal	/	/

**Notes.**

* Frozen storage (−20 °C/−80 °C) resulted in “precipitation + bimodal” for all three groups.

Based on the above results, the 1:1:2 formulation (DOPS/DOPC/DOPE = 1:1:2) that maintained monomodal distribution and excellent colloidal stability at 4  °C for 6 months, was selected as the optimal formulation for the present model.

#### Equivalence verification of dichloromethane substitution

**Figure 4:**
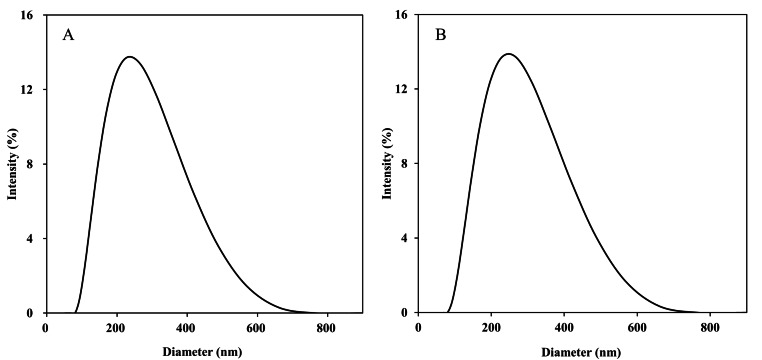
Particle size detection results of biomimetic membranes prepared with different solvents. (A) Particle size distribution (CH_2_Cl_2_). (B) Particle size distribution (CHCl3).

**Figure 5:**
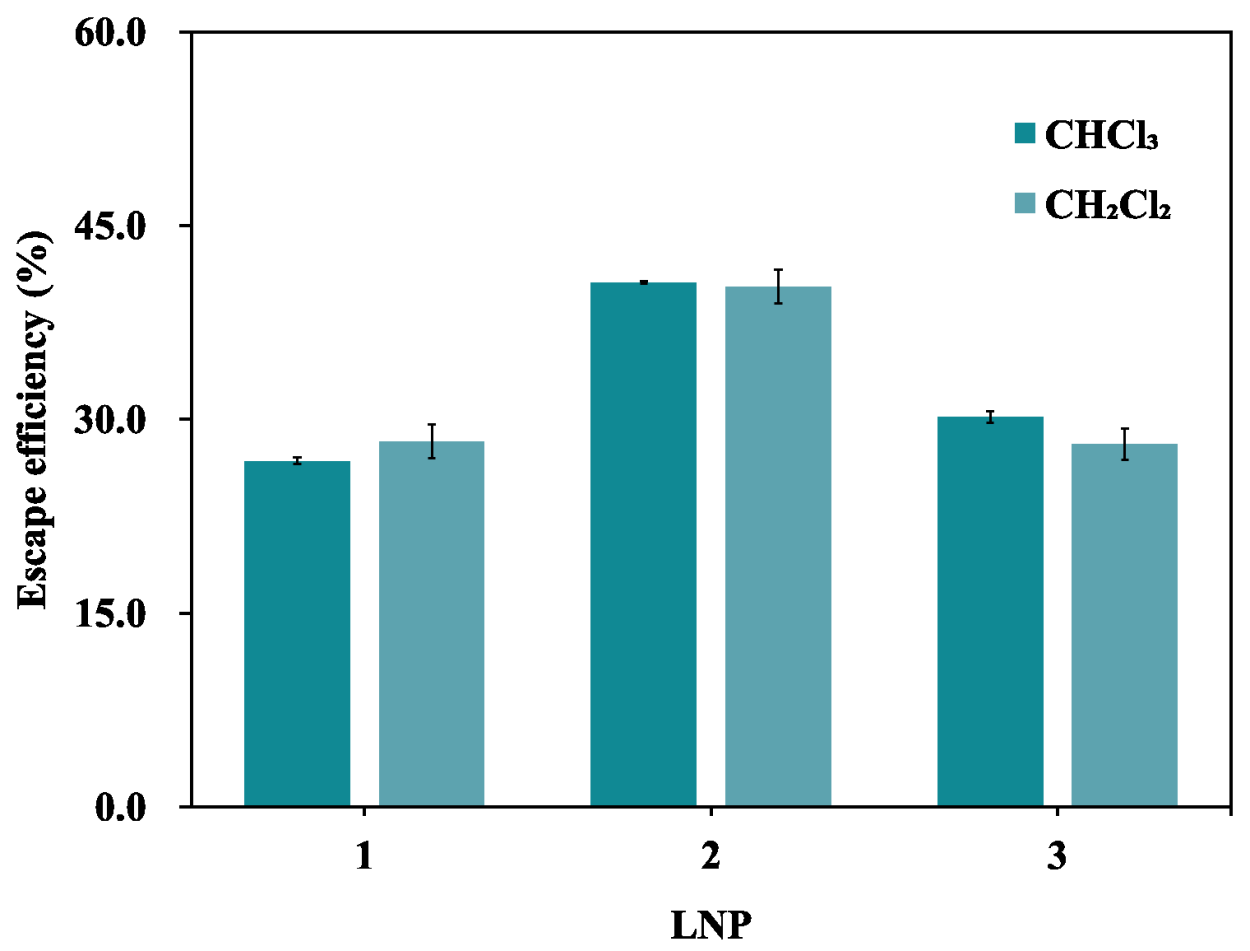
Comparison of escape efficiency results using biomimetic membranes prepared with different solvents (*n* = 2).

When preparing biomimetic cell membranes of the same formulation using the thin-film hydration method, the particle size (221.4 nm *vs.* 217.3 nm) and polydispersity index (PDI: 0.182 *vs.* 0.173) of the formulations obtained with dichloromethane (CH_2_Cl_2_) and chloroform (CHCl_3_) showed minimal differences (Fig. 4), indicating that solvent replacement did not cause particle aggregation or distribution heterogeneity. The escape efficiency test results for three independent batches of samples (Fig. 5) showed that the difference in escape efficiency between the CH_2_Cl_2_ and CHCl_3_ groups was ≤2.5%, with no systematic bias trend. Lipid content analysis (Table 2) indicated that the measured concentrations of each component in the CH_2_Cl_2_ group were closer to theoretical concentrations and had higher recovery rates, superior to the CHCl_3_ group. Visual observation of dissolution kinetics showed no significant difference in dissolution speed, completeness, or solution appearance between the two groups. Furthermore, the acute oral toxicity of dichloromethane is significantly lower than that of chloroform (rat LD_5__0_: 1,600–2,000 mg/kg *vs.* 908 mg/kg), and it is not subject to relevant regulatory constraints, fundamentally circumventing the toxicological and compliance risks of chloroform. In summary, dichloromethane was selected as an easily accessible, low-toxicity replacement for replace chloroform in all subsequent experiments.

#### Basis for elimination of vacuum drying step

In the optimization study of the biomimetic cell membrane preparation process, this study systematically evaluated the impact of the vacuum drying step on residual organic solvent levels. Experimental data showed (Table 3) that the dichloromethane residue level in samples without vacuum drying treatment was 0.60 µg/mL, decreasing to 0.31 µg/mL after 6 h of drying. According to ICH Q3C (R6), the residual limit for dichloromethane as a Class II solvent is 0.06% (equivalent to 600 µg/mL). The measured values for all experimental groups (0.31−0.60 µg/mL) were only 0.05%−0.1% of this limit, at a trace safety level. Moreover, the absolute removal amount after 6 h of drying (0.29 µg/mL) accounted for only 0.048% of the limit, providing no substantial gain in safety. Simultaneously, the vacuum drying process might induce changes in the orderliness of lipid molecules through thermodynamic stress, thereby damaging the assembly structure and phase stability of lipid molecules; thus, the risk associated with step outweighs the marginal benefit. Therefore, comprehensively considering process necessity, solvent safety thresholds, and protection of membrane integrity, the vacuum drying step was eliminated in the present model. This decision complies with ICH mandatory standards (residue level <<0.06%) and avoids potential damage to the membrane nanostructure from unnecessary processes, ensuring biomimetic membrane structural integrity and process robustness.

**Table 2:** Lipid content detection results of biomimetic membranes prepared with different solvents.

**Lipid component**	**Theoretical Conc. (mg/mL)**	**CH_2_Cl_2_**		**CHCl_3_**	
		**Measured Conc. (mg/mL)**	**Recovery (%)**	**Measured Conc. (mg/mL)**	**Recovery (%)**
DOPS	8.10	8.03	99.1%	7.63	94.2%
DOPE	14.88	14.06	94.5%	13.79	92.7%
DOPC	7.86	8.00	101.8%	7.46	94.9%

**Table 3:** Effect of vacuum drying time on solvent residue.

**Vacuum drying time (h)**	**Dichloromethane residue (μg/mL)**	**Percentage of pharmacopoeia limit (%)**
0	0.60	0.100%
1	0.33	0.055%
3	0.33	0.055%
6	0.31	0.052%

### Establishment and evaluation of the *in vitro* escape evaluation system

#### Validation of the pH-functional escape window

**Figure 6:**
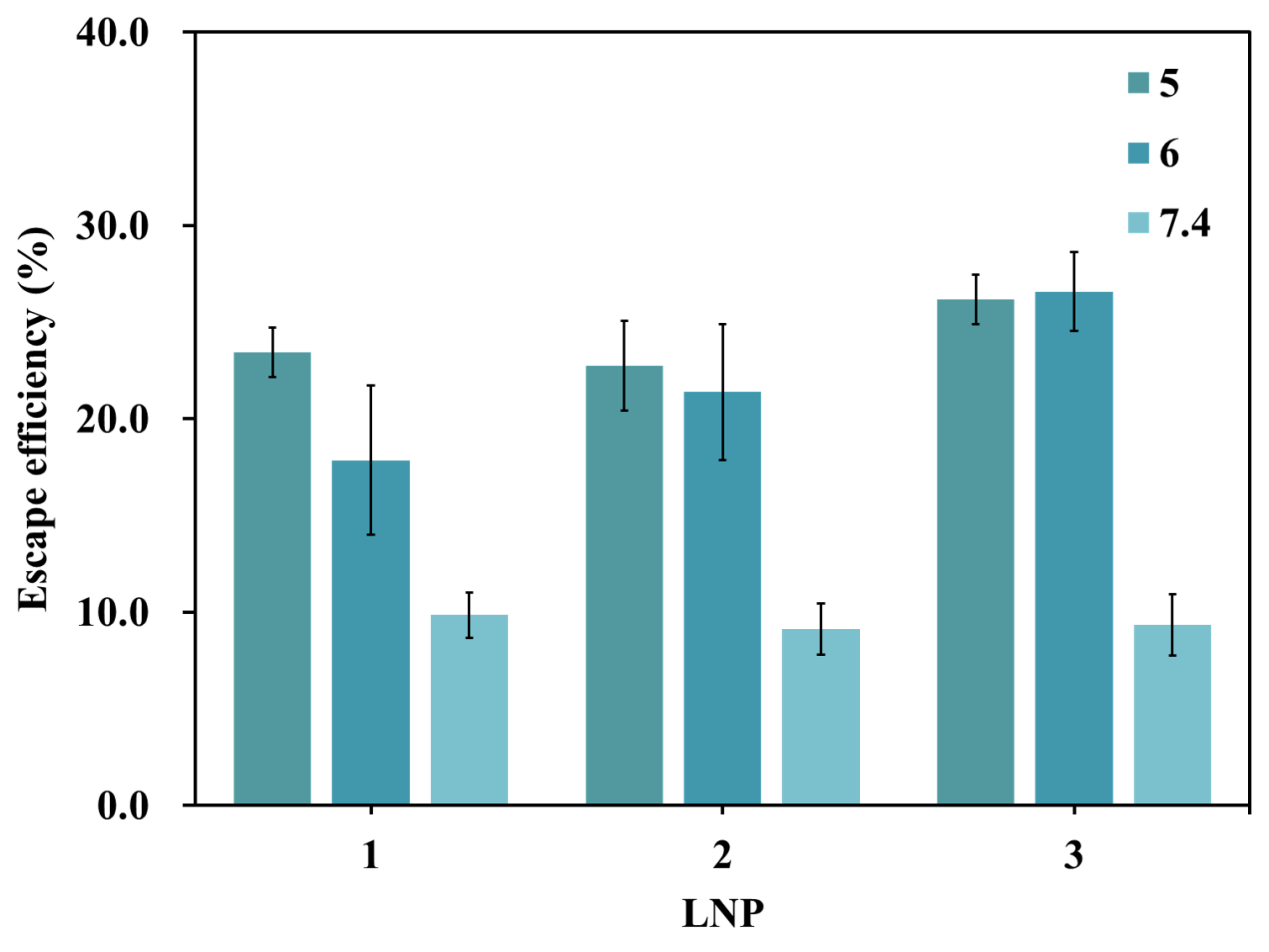
LNP-CpG ODN escape efficiency detection under different pH environments (*n* = 3).

Through quantitative evaluation of LNP endosomal escape efficiency under physiologically relevant pH environments—pH 7.4 (simulating extracellular/early endosome), pH 6.0 (critical escape period), and pH 5.0 (lysosomal degradation period)—marked differences were observed among three independent LNP samples (Fig. 6): the highest escape efficiency was measured under pH 5.0 conditions, followed by pH 6.0, while pH 7.4 showed the lowest escape efficiency. It is worth noting that the measured escape efficiency value peaked under pH 5.0 conditions. In such strongly acidic environment, the LNP undergoes self-assembly, causing partial free payload to be re-encapsulated, leading to an overestimation of the actual escape efficiency. However, under biological conditions (inside a cell), pH 5.0 corresponds to the lysosomal degradation phase, where the drug is prone to degradation and cannot effectively simulate the critical “escape” step, and therefore, pH 5.0 cannot be considered a relevant condition for the model. Accordingly, this study established the core environmental parameter for the *in vitro* escape evaluation model as pH 6.0, a condition that mimics more accurately the critical period for escape that occurs under physiological conditions (*e.g.*, late endosome to lysosome transition stage in the cell).

#### Correlation analysis between escape efficiency and TLR9 activation intensity

**Figure 7:**
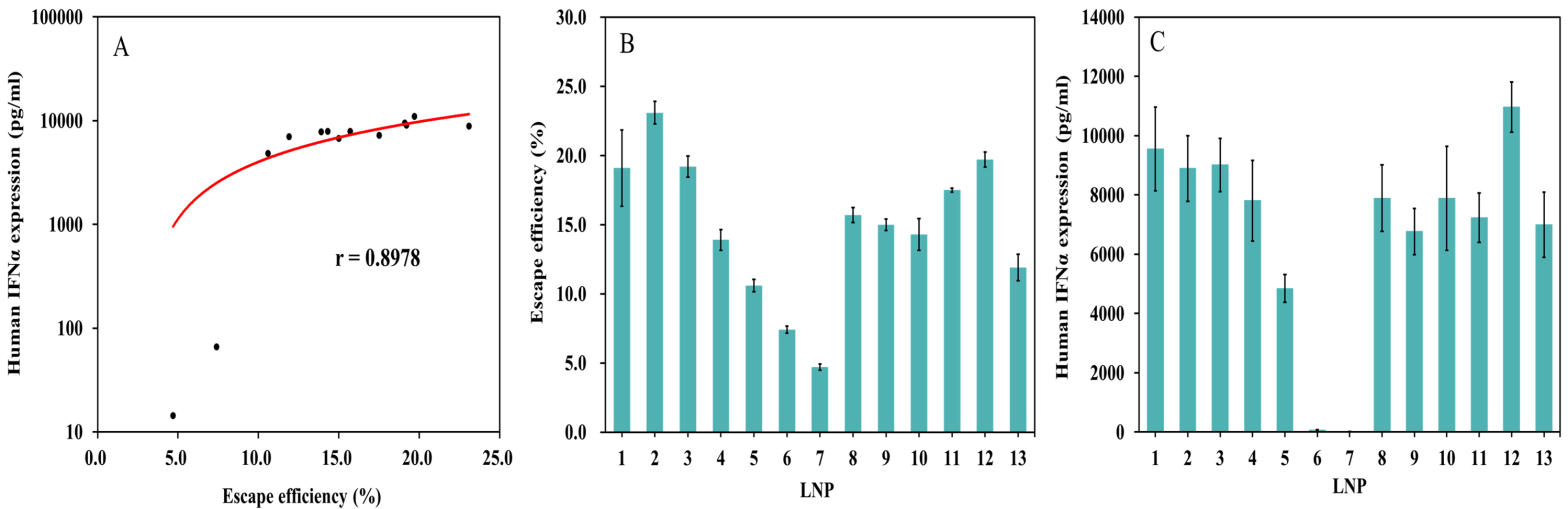
Correlation analysis between LNP-CpG ODN escape efficiency and TLR9 activation intensity. (A) Positive correlation between the escape effciency of 13 LNP samples and their mediated biological activity (*n* = 13). (B) Escape effciency in LNP-CpG ODN 13 LNP samples (*n* = 2). (C) TLR9 agonist activity in LNP-CpG ODN 13 LNP samples (*n* = 3).

Efficient intracellular escape is the key rate-limiting step for the release of nucleic acid payloads from endocytic vesicles into the cytosol, directly determining the activation efficiency of downstream biological effector pathways (Latz et al., 2004). A significant positive correlation was found between the *in vitro* escape efficiency and the mediated biological activity across the thirteen LNP samples (Pearson correlation coefficient *r* = 0.8978, 95% CI [0.6864–0.9693], ∗*p*∗ < 0.0001, Fig. 7A). The results indicated that an increase in escape efficiency (Fig. 7B) was accompanied by a systematic enhancement of biological activity (Fig. 7C), while a decrease in efficiency led to a significant decline in activity. Although some samples exhibited activity fluctuations at similar escape efficiencies—for example, the activity of two samples severely deviated from the main trend, later verified by mechanism to be caused by CpG motif disruption leading to nucleic acid payload inactivation—the overall data confirmed that escape efficiency can serve as a core indicator for predicting LNP biological activity. This provides an efficient *in vitro* prediction model for the rational optimization of LNP-based delivery systems.

## Discussion

For future perspectives, the methodological foundation laid here paves the way for incorporating advanced observational methods, such as Gal-8 reporter cell lines for visualizing endosomal membrane integrity and FRET-based real-time tracking of nucleic acid release kinetics. This would deepen the multidimensional understanding of the relationship between the *in vitro* model and the biological system, thus significantly increasing its analytical resolution and biological relevance.

Furthermore, the biomimetic membrane model established in this study, as a simplified system, presents certain inherent limitations. For instance, the model does not incorporate membrane proteins that may influence LNP fusion, lacks the asymmetric lipid distribution characteristic of natural membranes, and does not recapitulate the heterogeneous microdomain structures formed by cholesterol. While these simplifications are necessary for establishing a standardized screening platform, they also imply that our system cannot fully replicate the complexity of biological membranes. Future iterations of this platform could consider the incorporation of specific membrane proteins or asymmetric lipid arrangements to better simulate particular biological contexts.

Finally, during *in vivo* delivery, a protein corona rapidly forms on the LNP surface, a process that may significantly alter its interaction patterns with the endosomal membrane and subsequent immunological outcomes. Different protein components may modulate endosomal escape efficiency and the specificity of TLR9 pathway activation by altering the LNP’s surface properties, charge state, and recognition sites. Although the serum pre-incubation step introduced in our biomimetic platform preliminarily simulates this process, the regulatory mechanisms by which the precise composition and dynamic evolution of the protein corona govern delivery fate remain to be fully elucidated. This also highlights an important direction for the platform’s future development: constructing coronas with defined protein compositions could further elucidate the precise role of the protein corona in the intracellular trafficking and immune activation mediated by LNPs.

## Conclusions

This study successfully constructed an *in vitro* endosomal escape evaluation platform integrating serum protein corona simulation (simulating the initial stage of *in vivo* delivery), a biomimetic phospholipid bilayer barrier (accurately simulating endosomal membrane structure), and pH regulation (recreating the endosomal acidification process). This platform achieved precise quantification of LNP-CpG ODN endosomal escape efficiency. Through systematic optimization, the key parameters governing this process were determined. First, the optimal biomimetic membrane formulation was achieved using DOPS/DOPC/DOPE at a molar ratio of 1:1:2. This ratio significantly enhanced membrane fusion activity while maintaining high vesicle stability (polydispersity index PDI < 0.18), providing therefore an ideal membrane environment for efficient endosomal escape. Furthermore, through solvent optimization (using dichloromethane instead of chloroform) and process streamlining (eliminating the vacuum drying step), toxicity and compliance risks were significantly reduced, and potential damage to the membrane structure was avoided, thereby establishing a safe and efficient biomimetic membrane preparation workflow.

Next, the experiments confirmed that the pH 6.0 microenvironment, simulating late endosomes, was the critical node for LNP-CpG ODN to achieve efficient endosomal escape; whereas pH 5.0 (simulating the lysosomal environment) may lead to an overestimation of escape efficiency due to the risk of drug re-encapsulation and degradation.

Finally, the study found a strong positive correlation (*r* = 0.8978, *p* < 0.0001) between endosomal escape efficiency estimated by the developed model and the biological activity of the payload (innate immune response induced by the CpG ODN through TLR9 stimulation), and confirming therefore the predictive value of the model in the functional assessment and screening of LNP-based delivery systems.

In summary, this study constructed a simplified, controllable, and predictive *in vitro* evaluation tool, and more importantly, established a dual-track correlation evaluation framework linking *in vitro* escape efficiency and biological activity. This framework provides solid theoretical support and practical guidance for the rational design (*e.g.*, screening novel ionizable lipids or pH-sensitive materials), formulation process optimization, and preclinical efficacy prediction of LNP-CpG ODN delivery systems, promoting the development of efficient CpG ODN adjuvant delivery strategies.

##  Supplemental Information

10.7717/peerj.21147/supp-1 Supplemental Information 1 Raw data

10.7717/peerj.21147/supp-2 Supplemental Information 2 Gas chromatographic analysis of dichloromethane residues in samples subjected to 0-6 h vacuum drying (A) 0h GC residue - first injection chromatogram. (B) 0h GC residue - second injection chromatogram. (C) 1h GC residue - first injection chromatogram. (D) 1h GC residue - second injection chromatogram. (E) 3h GC residue - first injection chromatogram. (F) 3h GC residue - second injection chromatogram. (G) 6h GC residue - first injection chromatogram. (H) 6h GC residue - second injection chromatogram.

## Additional Information and Declarations

### Data Availability

The following information was supplied regarding data availability: The raw measurements are available in the Supplemental File.
