# Supplementary figures and images for: A biomimetic membrane platform for predictive screening and rational engineering of endosomally escaped lipid nanoparticle-CpG oligodeoxynucleotides delivery systems
Source: PeerJ. 2026 May 5;14:e21147. doi: 10.7717/peerj.21147 (PMC13155235; doi:10.7717/peerj.21147)

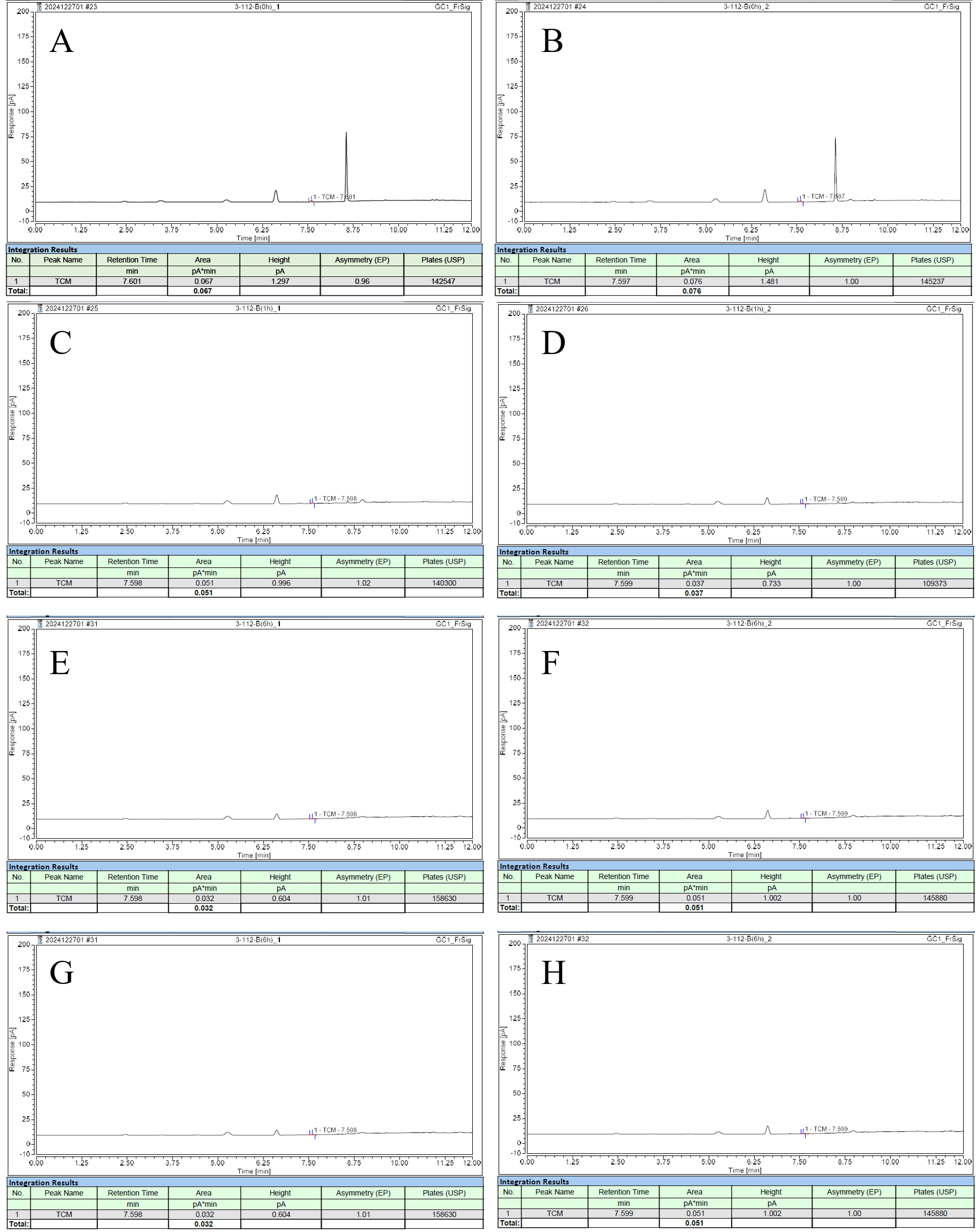

Supplement: Supplemental Information 2 — (A) 0h GC residue - first injection chromatogram. (B) 0h GC residue - second injection chromatogram. (C) 1h GC residue - first injection chromatogram. (D) 1h GC residue - second injection chromatogram. (E) 3h GC residue - first injection chromatogram. (F) 3h GC residue - second injection chromatogram. (G) 6h GC residue - first injection chromatogram. (H) 6h GC residue - second injection chromatogram. [file peerj-14-21147-s002.png]
